# A thematic analysis of the role of the organisation in building allied health research capacity: a senior managers’ perspective

**DOI:** 10.1186/1472-6963-12-276

**Published:** 2012-08-27

**Authors:** Xanthe Golenko, Susan Pager, Libby Holden

**Affiliations:** 1 School of Medicine Logan campus Griffith University, University Drive Meadowbrook, Meadowbrook, Q 4131, Australia; 2 Metro South Health, Level 2, 7 Clunies Ross Court, Brisbane Technology Park, Eight Mile Plains, Q 4113, Australia; 3 School of Population Health, University of Queensland, Herston Road Herston, Q Australia

**Keywords:** Research capacity building, Organisational role, Allied health

## Abstract

**Background:**

Evidence-based practice aims to achieve better health outcomes in the community. It relies on high quality research to inform policy and practice; however research in primary health care continues to lag behind that of other medical professions. The literature suggests that research capacity building (RCB) functions across four levels; individual, team, organisation and external environment. Many RCB interventions are aimed at an individual or team level, yet evidence indicates that many barriers to RCB occur at an organisational or external environment level. This study asks senior managers from a large healthcare organisation to identify the barriers and enablers to RCB. The paper then describes strategies for building allied health (AH) research capacity at an organisational level from a senior managers’ perspective.

**Methods:**

This qualitative study is part of a larger collaborative RCB project. Semi-structured in-depth interviews were conducted with nine allied health senior managers. Recorded interviews were transcribed and NVivo was used to analyse findings and emergent themes were defined.

**Results:**

The dominant themes indicate that the organisation plays an integral role in building AH research capacity and is the critical link in creating synergy across the four levels of RCB. The organisation can achieve this by incorporating research into its core business with a whole of organisation approach including its mission, vision and strategic planning. Critical success factors include: developing a co-ordinated and multidisciplinary approach to attain critical mass of research-active AH and enhance learning and development; support from senior managers demonstrated through structures, processes and systems designed to facilitate research; forming partnerships to increase collaboration and sharing of resources and knowledge; and establishing in internal framework to promote recognition for research and career path opportunities.

**Conclusions:**

This study identifies four key themes: whole of organisation approach; structures, processes and systems; partnerships and collaboration; and dedicated research centres, units and positions. These themes form the foundation of a model which can be applied to assist in achieving synergy across the four levels of RCB, overcome barriers and create an environment that supports and facilitates research development in AH.

## Background

### Research capacity building

Research capacity building (RCB) in primary health care (PHC) has become an important focus for health services internationally. RCB can be defined as a “process of individual and institutional development which leads to higher levels of skills and greater ability to perform useful research” [1]. The primary aim of RCB initiatives is to increase the research base of health care professionals and organisations, and close the gaps between research, policy and practice [2,3].

The literature describes RCB as complex, multi-level and multi-factorial [2,4,5]. Cooke, 2005 proposes that RCB functions across four levels; individual, team, organisational, and supra-organisation (networks and support units). RCB at an individual level focuses on skills development, mentoring and engaging clinicians in research, the team level emphasises a team-based approach to achieve the appropriate mix of skills and enhance knowledge sharing. At an organisational level RCB relates to building elements of research sustainability and continuity, overcoming barriers and enhancing research culture. At the supra-organisational level the focus is on establishing partnerships with stakeholders and supporting linkages, providing access to funding and increasing appropriate dissemination. RCB interventions are generally targeted at specific levels however Cooke, 2005 argues that “one level can have an impact on capacity development at another level, and could potentially have a synergistic or detrimental effect on the other” [4]. This approach suggests that each level of RCB is equally important and interdependent.

RCB measures have traditionally focused on individual level research skills such as generating research ideas, writing research protocols and analysing and interpreting results [6] or outputs including publications, conference presentations, successful grant applications and qualifications [4]. There is growing support however for a more holistic approach [3,4,7,8]. Farmer and Weston, (2002) propose a conceptual model for RCB based on the following six principles: RCB should influence all levels in a ‘whole system’ approach, accommodate diversity, reduce barriers, enable collaboration, provide feedback and mentoring, and facilitate a networking process. They suggest that organisations can create an environment where research is valued, expected and enjoyed by explicitly valuing continuous learning and research, developing centres of research excellence, supporting the development of independent investigators, creating an atmosphere of mutual respect and appreciation for the contribution of all members, and supporting multi-disciplinary and multi-method approaches [7].

While theoretical frameworks to evaluate RCB across the four levels have recently been developed [4,7], few studies have been undertaken to measure the effectiveness of RCB interventions that incorporate all four levels including organisation and external environment level measures [9].

### Allied health and research capacity building

Allied health (AH) is an overarching term used to describe a diverse group of healthcare professions, each with their own unique focus [10,11]. These include physiotherapy, speech pathology, occupational therapy, dietetics and psychology. In Australia, AH represent around 20% of health professionals, and have recently experienced higher growth rates than both medical and nursing. AH professionals work across public and private sectors, providing services through acute hospitals, specialist services, community health, primary care, aged care and non-health settings [12]. AH work often requires a multi-disciplinary and holistic approach to complex interventions [11].

It has been identified that a strong evidence base is lacking in AH [11], yet AH professionals are faced with increasing pressure to ensure their practice is evidence-based as demand increases for rehabilitation and intermediate care [13]. Many AH academic disciplines are relatively new [10], therefore many AH professionals lack skills and knowledge in producing and using research [2], and few AH professions have sufficient numbers of research-active practitioners to generate a sound research evidence base [9,14]. In addition, AH professionals are faced with heavy case-load demands [11], and there is little opportunity for developing research skills or combining research and clinical careers [15].

### Purpose of the research

Existing literature presents a broad theoretical perspective of RCB in PHC however a better understanding of the role organisations play can assist in achieving synergy across the four levels of RCB, overcome barriers and improve the effectiveness of RCB initiatives in the AH context.

This paper describes and analyses AH senior manager perspectives of how organisational factors impact on RCB. Based on the research findings, a thematic model is presented, which identifies elements perceived to promote and enhance research culture. We then identify four key issues emerging from this study and discuss how the model can be applied to address these issues and assist in creating an environment that supports and facilitates RCB in AH.

## Methods

### Research design

This research is a qualitative component of a larger study to evaluate the effectiveness of a settings-based approach to developing research capacity in individual staff members and research culture in AH teams. The parent research project was a non-randomised matched-pairs trial design. It compared the impact of a multi-strategy intervention for five allied health teams recruited from a metropolitan health services district with five control teams from outside the district. Intervention and control teams were matched on service role and approximate size. Changes in mean scores between pre and post intervention were compared with controls across three domains: individual, team and organisational. Individual team members and team leaders completed a self-reported written survey pre and post intervention, and team leaders and senior managers for each team were interviewed pre and post intervention to provide contextual information regarding potential factors that could impact on the outcomes of the intervention. An evaluation of the parent research project can be found in a paper by Holden et al. (2012), “Evaluating a team-based approach to research capacity building using a matched-pairs study design” [16]. However the scope of this paper is confined to the interviews conducted with senior managers at pre-intervention.

### Interview questions

The interview questions, shown in Table 1, were developed from the literature in three key areas: RCB, organisational management and AH. The questions are focused around four main themes based on the literature: 1. Changes in the organisation’s external and internal environments that have impacted on AH research and AH generally [1-5,7-9]; 2. Organisational support for research in AH [3,4,7-9,14,16-21]; 3. Barriers and motivators for AH research and 4. Critical success factors for strong research culture [2,9-11,13-16]. Participants were also asked to describe their role within the organisation and their experience with research in order to gauge their level of expertise and understanding of research, the organisation and AH.

**Table 1:** Interview questions

1.1. Please describe your role / function in the organisation	
1.2. Please describe your experiences with research	
1.3. Please describe any recent events or major changes within the organisation that have impacted on Allied Health generally or Allied Health research in particular	
1.4. Please describe any recent external events or major changes that have impacted on Allied Health generally or Allied Health research in particular	
1.5. What research structures/supports/processes currently exist within your organisation for Allied Health?	
1.6. What do you see as the biggest barriers to research for Allied Health?	
1.7. What do you see as the biggest motivators for research for Allied Health?	
1.8. What do you consider to be the critical success factors in establishing a strong research culture in this organisation?	

### Sampling and recruitment

A purposive sampling method was used to recruit the senior managers; participants were selected based on their direct line management of the teams recruited into the parent study. This involved recruiting all allied health senior managers from the health district where the five intervention teams were based and allied health senior managers responsible for each of the five control teams in surrounding health districts. One of the senior managers was responsible for two of the teams in the study. Therefore a total of nine senior managers from five metropolitan districts within the organisation were recruited.

Informed consent packages were emailed to participants and written, signed consent forms were obtained from all participants prior to conducting the interview. Ethics approval was obtained from the following Human Research Ethics Committees (HREC): Griffith University HREC MED/10/08/HREC, The Prince Charles Hospital Metro North Health Service District HREC/09/QNRC/8, West Moreton Health Services District HREC/09/QWMS/1, Metro South Health Services District HREC 2008/217, and Children’s Health Services District HREC/09/QRCH/70.

### Data collection and analysis

Data was collected between March and May 2009. A set of eight interview questions was emailed to participants at least one week prior to the interview to allow time for reflection. Semi-structured interviews were conducted by an experienced qualitative researcher and the duration of the interviews was approximately 30 minutes. All participants were asked the same eight questions to guide discussion and provide a broad structure to allow for comparison across the sample. This also made it possible to compare individual responses from baseline with follow up interviews to identify changes that may have occurred within the time frame. The questions were open-ended and the semi-structured interview method provided scope for the interviewer to ask for further explanation or clarification and to explore and discuss various topics identified by participants from their perspective.

The interviews were recorded and detailed notes were taken by the interviewer. The recordings were then transcribed by a research assistant. Once all of the interviews were completed; the transcriptions, recordings and notes were then utilised for the analysis which was carried out between June 2009 and February 2010. Qualitative research analysis software NVivo was used to conduct a conceptual analysis and code the interviews by grouping common topics and issues, and categorising them under labels which represented particular themes that emerged. A relational analysis was then conducted using NVivo to examine relationships between the themes that were derived from the data. A highly complex model was developed based on this analysis, which was then simplified to form the “*Thematic model for RCB in AH at an organisational level”* as presented in this manuscript. All interviews were coded and analysed by the one researcher for consistency, however throughout the analysis period, the authors reviewed the coding, themes and model development using an iterative, consensus decision-making process for category reliability.

## Results

### Participants

Of the nine participants selected, three were District Executives, and three were Division Chairs, there was one Director, one Executive Director, one Team Leader, and two of these were also members of Clinical Councils. All participants had professional and / or operational responsibilities for AH.

All participants managed teams of staff in hospital and / or community based services and units, including child and youth services, aged care, acute and palliative care, mental health promotion, injury prevention and physical activity. The majority of participants reported having little research experience themselves other than that acquired from undergraduate or post graduate studies. One participant was undertaking a PhD at the time of the interviews.

### Research findings

Factors operating both externally and internally to the organisation were perceived by all participants as affecting the internal climate and culture and having substantial influence over the organisation’s RCB capabilities. External factors were primarily perceived as barriers, while several internal factors were identified as enablers.

External factors that were perceived as barriers include increased clinical workloads associated with the growing and aging population. Workforce shortages and work-life balance issues such as juggling family responsibilities were cited as reasons why it is difficult for clinicians to take time “off-line” to do research or to participate in RCB activities.

"*“Many of the allied health professions are female so when people are outside of work they actually have a whole different work place when they go home in terms of being a parent - significant family responsibilities. So time is a huge issue for people.”* (Participant 2)"

"“*We are very thin on the ground, we have huge case load demands and we have huge waiting lists. So staff are very clinically focused, and to be able to step out of that to come off line to do research actually requires them to be able to access some kind of funding … it is not possible to do it in conjunction with your routine work.”* (Participant 3)"

Some managers also cited other external factors such as a lack of recognition and priority for AH research amongst the scientific community and funding bodies compared to other professions such as medicine and nursing. These were perceived as significant barriers, resulting in a lack of funding for projects and restricted research dissemination.

"*“I think that a barrier to research in allied health is there is probably not the same emphasis or importance given to research in allied health interventions as there are for medical kinds of interventions like drug trials … you can see this right through the institutional structure of research.”* (Participant 1)"

**Figure 1:**
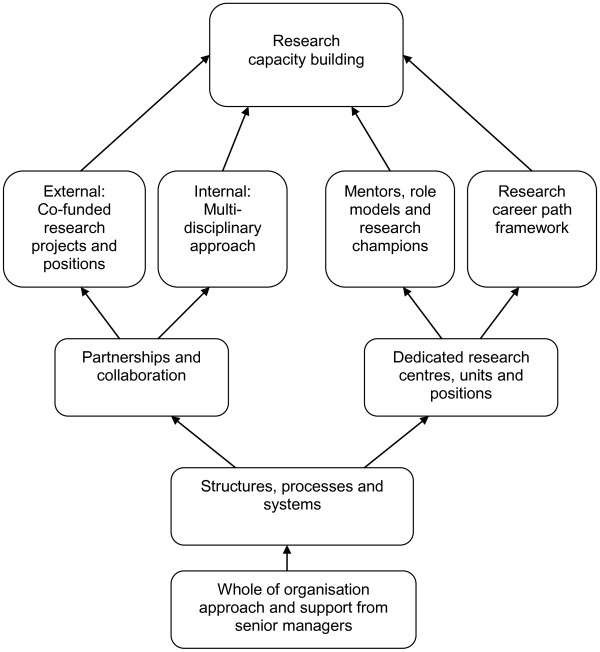
Thematic model for RCB in AH at an organisational level.

Analysis of the data also identified a number of internal factors perceived as enablers in facilitating RCB in AH. These factors have been grouped into four dominant themes and form the basis of a thematic model for RCB in AH at an organisational level described below (see Figure 1).

#### Theme 1: Whole of organisation approach and support from senior managers

Participants identified that adopting a whole of organisation approach to RCB can assist in strengthening research culture within the organisation. Similarly, high importance was placed on creating an environment to support research which requires a co-ordinated approach, organisational understanding, having governance structures in place, appropriate incentives to do research, and recognition for research as a career path.

"*“Research needs to be core business, so it actually needs to be built in as an expectation as to what every clinician does.”* (Participant 6)"

"*“For me it is about having the governance structures in place and to give coordinated support to make sure it happens appropriately. Then it is about establishing what are the incentives to have staff participate in the research, and one is that it benefits your career path, that will motivate people secondly that you are supported from a staff time perspective to do it as well, recognition, a capacity issue. You could have any one of them by themselves but for the most significant impact in research you really need those three things happening together.”* (Participant 4)"

Participants also emphasised the importance of infrastructure, access to resources and strong leadership support from senior managers;

"*“I think this is impressing upon people that this is part of what we do, having that infrastructure to support it having the learning and development behind it to support it, being able to access library resources and those sorts of things and having those champions.* (Participant 5)"

"*“The organisation can be supportive in terms of all its policies and procedure, but if the line managers don’t support it, it all falls apart.”* (Participant 9)"

"*“I think two key things (critical success factors) for me would be you need to build it into people’s jobs or at least some people’s jobs amongst the senior staff and you need to have good leadership support because from then all things will flow.”* (Participant 1)"

#### Theme 2: Structures, processes and systems

Organisational structures, processes and systems were identified as having a strong influence on RCB in AH. All participants discussed recent industrial changes and the move toward a new award structure within the organisation. Some saw this as having positive effects on AH research, for example, new opportunities for AH to develop research skills through scholarships and workforce development programs, increased incentives to acquire research qualifications, and recognition for research.

"*“That is the other major success factor is the (new) award, providing that structure for people who want to do research.* (Participant 3)"

However, others felt that not all sites benefited, and that the process of re-evaluating award rates was time consuming and created conflict amongst staff.

Participants also discussed a recent structural change within the organisation which saw an amalgamation of some districts. This created opportunities for collaboration, assisted in building critical mass in some professions, and linked some areas with others that had a stronger research culture.

"*“Through the amalgamation we created this (conjoint) position so we have actually got better structure and line management support for allied health.”* (Participant 7)"

Streamlining processes such as applying for ethics, and developing systems that allow clinicians to take time off line to do research were also perceived as important enablers for research capacity building.

"“*I think one of the things that will be really positive is when they do finally have single HREC’s. It is really frustrating to have to go through so many HREC’s.”* (Participant 6)"

"*“(Building in time) is not typical in allied health professions, so I think that would be a powerful motivator and powerful enabler”* (Participant 1)"

#### Theme 3: Partnerships and collaboration

Participants believed that forming strong external partnerships with other organisations, in particular Universities, can assist in providing AH with access to experienced researchers, research skills training and opportunities to apply research skills, access to infrastructure and resources such as libraries and computer software, and access to funding. Partnerships were primarily established through collaborative research projects and jointly funded research positions.

"*“Positions that are discipline specific have provided the infrastructure and support for the disciplines to do more research.”* (Participant 3)"

"*“We are doing a joint research project with the University and that has been really positive, it gives us access to their equipment and we get the research we want done.”* (Participant 6)"

Internal links and collaboration between community and hospital settings as well as between the different professions were considered important enablers for research in AH. Managers perceived that collaboration can assist with developing a co-ordinated approach to research rather than individual research activities occurring in isolation, increase critical mass in some professions, and facilitate the transfer of knowledge between units through learning circles (journal clubs) and developing learning packages to pass on to other units.

"*“I think there has been improved coordination in that it is not individual research activities that are occurring in isolation of each other, it is more of a coordinated approach. That has been one of the big things which has assisted with allied health research.”* (Participant 4)"

"*“If you have evidence-based journal clubs or groups within your organisation, then it means that people who have interest but perhaps not any experience, can spend time with people who do know how to do it and be encouraged.”* (Participant 1)"

Some managers thought that while a multidisciplinary approach to research is critical, they also acknowledged the importance of ‘profession- specific’ research.

#### Theme 4: Dedicated research centres, units and positions

Participants identified that recent reforms within the organisation resulted in funding being allocated to establish a number of dedicated research centres, units and positions including an allied health research centre, ethics and evidence based practice (EBP) committees, advisory and clinical governance units and research officers. These were described by all participants as having a positive impact on research in AH and were important motivators for staff, providing opportunities for career advancement in research and attracting experienced researchers to mentor and drive research.

"“*Now we have got an the allied health advisory unit, which does do some supports for research and that’s where the grants are funded through and provides strategic advice on a state-wide level which is good, that is a positive thing and I know that they have got a research steering committee that runs out of there so that is a big picture thing.”* (Participant 6)"

"*“The Award has an impact on the level of research that occurs and formally recognises research in the career structure which is an additional incentive that wasn’t there before in allied health.”* (Participant 4)"

Managers acknowledged however, that support for research should not be restricted by or limited to these research specific positions and suggested that research support needs to be extended to other clinicians who are interested in research and linking them throughout the organisation.

## Discussion

The results emerging from this study provide insights into how a variety of organisational factors can influence research capacity within AH. The findings strongly suggest that organisations play a critical role in RCB in AH and that external events and circumstances as well as internal culture and climate influence research capacity in AH. The resulting thematic model focuses on internal factors and identifies four key themes which provide a foundation for organisations to develop strategies for building research capacity in AH: 1. Whole of organisation approach and support from senior managers; 2. Structures, processes and systems; 3. Partnerships and collaboration; and 4. Dedicated research centres, units and positions. These findings are consistent with current literature and provide empirical evidence to support the theories and concepts proposed by other researchers in the field [4,7,9,11,22].

Findings from this study also identify four key challenges associated with RCB in AH. These are: lack of time and financial resources; lack of recognition for AH research amongst the scientific community; the complexity of multi-disciplinary, team-based research in AH; and lack of a career path for research in AH. This discussion will focus on how organisations can apply the *Thematic Model for RCB in AH at an Organisational Level* to address these challenges and improve RCB outcomes by achieving synergy across the four levels of RCB.

### Challenge 1: Lack of time and financial resources

This study found that lack of time and financial resources create the most significant barriers to RCB in AH. This finding is consistent with the literature across the primary health care sector and highlights the enormous challenge faced by healthcare organisations in satisfying the increasing demand for quality healthcare under constrained budgets and limited resources [17,18]. As demand for healthcare increases, so do competing pressures from stakeholders on AH. While organisations strive for improved efficiencies, AH professionals strive to provide quality care to patients drawing on evidence to improve practice and health outcomes [18]. In addition AH place high importance on work-life balance, career development, job satisfaction and recognition [17]. This finding suggests a misalignment between the operational goals of the organisation and the professional goals of AH.

*Theme 1* of the *Thematic Model for RCB in AH at an Organisational Level* suggests that a whole of organisation approach can assist in achieving better alignment between operational and professional goals. This can be achieved through greater internal consistency; ensuring that research is built into the organisation’s mission and vision, developing strategies to support the overall objectives of the organisation, designing RCB activities to maximise effective and efficient use of resources and evaluating research processes and outcomes. This finding is supported by the literature which suggests there is a lack of recognition for PHC research as an institutional, organisational and professional core value [18,22]. A “whole of organisation” approach has been recognised as effective in building research capacity [9,14,19] by adopting a culture of patient driven care and evidence-based practice [10], overcoming barriers to research such as lack of infrastructure, payment structures that do not compensate for research activity [7,20], and accommodating diversity and differences in research interests, professional backgrounds and clinical practices [21].

*Themes 1 and 2* of the model also suggest that support from senior managers demonstrated through establishing structures, processes and systems specifically designed to overcome barriers and create an environment that supports research are critical in building research capacity. This finding is supported by Caldwell et al., (2008) which suggests that managers have a responsibility to implement structures and systems that will support practitioners to do research, and support and facilitate participation in research activity [21].

### Challenge 2: Lack of recognition for AH research amongst scientific community

This study has found evidence to suggest a lack of recognition for AH research amongst the scientific community including funding bodies, Universities and academic journals resulting in limited access to funding, and low levels of research output, dissemination and publication. AH research may be perceived as poor quality because AH professions are relatively new academic disciplines therefore very few AH professionals are at doctorate level and research training provided in health settings is inadequate for the type of research expertise expected by academic institutions. In addition, there are few opportunities for practitioners to combine research and clinical careers [15]. This has resulted in limited research awareness, and lack of capacity and capability including skills such as research design and writing for publication amongst AH [10,11,15].

Evidence from the literature suggests that funding allocation for AH research is low and imbalanced compared to that of other professions internationally [11,15]. Health resource allocation favours hospitals and specialist care [9], and funding for research is directed towards high quality, randomised control trials often conducted in multiple sites and undertaken by experienced researchers in priority driven areas [15]. It is extremely difficult therefore for AH to attract funding under current systems [11,15]. NHS Scotland (2004) considers that AH produce high quality patient focused research, but acknowledges their influence on the wider health care sectors has not yet been achieved [10]. It also argues that AH are well positioned to inform research priorities and identify areas where research funding should be targeted as they make up a significant proportion of staff in direct patient care [11,14].

*Theme 3* of the *Thematic Model for RCB in AH at an Organisational Level* suggests that partnerships and collaboration, in particular with Universities can assist in improving the perception of AH research amongst the scientific community. These findings are supported by current literature which suggests that partnerships through conjoint positions can provide access to experienced researchers, research expertise, research skills training and opportunities to apply research skills. Collaborative research projects can assist in providing access to additional funding opportunities, as well as resources and infrastructure. Collaboration and jointly funded positions are suggested to encourage freer movement of practitioners and academics between applied and theoretical areas of work and encourage post-graduate education which is important in gaining recognition amongst the wider health care sectors [11,15,22].

### Challenge 3: Multi-disciplinary and team-based research in AH

Findings from this study indicate that there is increasing emphasis on multi-disciplinary and team-based approaches to research in the primary care setting. However, the fragmented nature of healthcare organisations, complex environment of primary healthcare and characteristics of AH professions with their own unique focus have resulted in a lack of critical mass of research active professionals in AH [9]. In addition, AH are often geographically dispersed and juggling heavy case-load demands [11] which also contribute to the challenges of conducting multi-disciplinary research [2,9,11].

*Theme 3* of the *Thematic Model for RCB in AH at an Organisational Level* found that internal partnerships and networks can help to overcome these challenges. This finding is supported by Farmer and Weston, 2002 which suggests internal partnerships that enable collaboration between researchers and professional groups and especially multi-disciplinary teams can assist in building critical mass, facilitate knowledge transfer and enhance organisational learning. They can also improve efficiencies by sharing resources and infrastructure [7].

### Challenge 4: Career path for research in AH

Findings from this study are consistent with current literature which suggest a lack of career path opportunities for research in AH [10,18]. *Theme 4* of the *Thematic Model for RCB in AH at an Organisational Level* found that establishing dedicated research centres, units and positions can assist in addressing this issue by enabling the organisation to establish a research career path framework and provide remuneration and career development opportunities, which are important in fostering AH researchers’ development and their retention in the organisation [10].

Research centres and units can assist in providing necessary infrastructure to achieve research sustainability [13,22], while dedicated research positions enable the organisation to strengthen research leadership through mentoring, role models and research champions who can drive research and facilitate research dissemination. Shaw, 2004 suggests that enthusiastic and commited individuals can drive research through their ability to influence others and co-ordinate activities and teams across departmental and organisational boundaries [23]. Mentoring has been identified as a key element in training and development and important in providing support to young researchers so they can develop research skills before they are overwhelmed by other demands [11,22].

### Limitations

There are three key limitations associated with this study design due to the fact that it was a qualitative component of a larger study to evaluate the effectiveness of a settings-based approach to developing research capacity in individual staff members and research culture in AH teams. Firstly, only nine participants were recruited because there were nine teams recruited into the parent study and the interviews were conducted with the senior manager who was responsible for each of these teams. Due to the restricted number of participants in this sample, thematic saturation may not have been achieved with such a small sample. However, given that all the relevant AH senior managers from one health service district within the organisation were interviewed along with an AH senior manager from each of the four other surrounding health service districts, it is likely that this is an adequate sample to explore the topic of organisational factors influencing AH research activity and culture within this organisation.

A further limitation of this study is that all the participants are from one large healthcare organisation and participants are speaking from their own perspective and not on behalf of the organisation, therefore generalisations should be made with caution. While the organisational factors identified by senior managers that impact on RCB are specific to this type and size of organisation and AH context, key themes may be extrapolated and tested for relevance in other organisations facing similar issues as identified in this discussion.

Finally, this study design also has limitations associated with the analysis being conducted by a different person to the interviewer and all interviews being completed prior to commencing analysis. This process did not allow for the interviewer to return to the participants for content verification and ongoing analysis. However, as part of the parent study, follow-up interviews were conducted 12 months later with the same participants using the same interview questions and semi-structured interview method. The follow-up interviews were conducted to identify any changes that may have occurred either internally or externally to the organisation and to assess any confounding affect they may have on individual and team research capacity and culture. The findings from the follow up interviews are not within the scope of this manuscript, however they are discussed in a paper by Holden et al. (2012), *“Evaluating a team-based approach to research capacity building using a matched-pairs study design”*[16]. Findings revealed that key themes identified from baseline interviews were consistent with follow-up interviews and a number of strategies to develop research capacity which were identified in the first set of interviews, were being developed and adopted by the organisation and indicating positive outcomes.

## Conclusions

This study aims to explore the role of the organisation in building AH research capacity from a senior manager’s perspective. Findings from this study suggest that the organisation is in a strong position to influence the research capacity of AH and their ability to use research to inform practice. Organisations can be seen as a critical link in creating synergy across the four levels of RCB to overcome barriers and effectively build research capacity. A whole of organisation approach can assist in developing an environment and culture that supports research. Promoting research as an organisational core value and support from senior managers can be demonstrated through establishing structures, processes and systems to facilitate research. External partnerships and collaboration can help to improve the perception of AH research amongst the scientific community by providing AH with access to research skills training, expertise and funding through collaborative research projects and positions. Internal partnerships assist in building a critical mass of research active AH, knowledge sharing and organisational learning and efficient use of resources. Finally, dedicated research centres, units and positions can provide AH with career path opportunities and mentors and strengthen research leadership within the organisation.

## Competing interests

The authors declare that they have no competing interests.

## Authors’ contributions

SP participated in the design of the study, development of the interview questions, co-ordination of the interviews, reviewed coding, emerging themes and model development, and assisted in drafting and reviewing the manuscript. LH was instrumental in the design of this study, development of the interview questions, reviewed coding, emerging themes and model development, and contributed to drafting the manuscript and critically revising it. All authors read and approved the final manuscript.

## Pre-publication history

The pre-publication history for this paper can be accessed here:

http://www.biomedcentral.com/1472-6963/12/276/prepub
